# Evaluation of telehealth support in an integrated respiratory clinic

**DOI:** 10.1038/s41533-022-00304-9

**Published:** 2022-11-11

**Authors:** Lauren Fox, Emily Heiden, Milan A. J. Chauhan, Jayne M. Longstaff, Lara Balls, Ruth De Vos, Daniel M. Neville, Thomas L. Jones, Anthony W. Leung, Lydia Morrison, Hitasha Rupani, Thomas P. Brown, Rebecca Stores, Anoop J. Chauhan

**Affiliations:** 1 Portsmouth Technology Trials Unit, Portsmouth Hospitals University NHS Trust, Portsmouth, UK; 2 Respiratory Medicine, Portsmouth Hospitals University NHS Trust, Portsmouth, UK; 3 Bordon Medical Centre, Bordon, UK; 4 Faculty of Science and Health, University of Portsmouth, Portsmouth, UK

**Keywords:** Health services, Asthma, Chronic obstructive pulmonary disease, Outcomes research

## Abstract

Supporting self-management is key in improving disease control, with technology increasingly utilised. We hypothesised the addition of telehealth support following assessment in an integrated respiratory clinic could reduce unscheduled healthcare visits in patients with asthma and COPD. Following treatment optimisation, exacerbation-prone participants or those with difficulty in self-management were offered telehealth support. This comprised automated twice-weekly telephone calls, with a specialist nurse triaging alerts. We performed a matched cohort study assessing additional benefits of the telehealth service, matching by: confirmed diagnosis, age, sex, FEV_1_ percent predicted, smoking status and ≥1 exacerbation in the last year. Thirty-four telehealth participants were matched to twenty-nine control participants. The telehealth cohort generated 165 alerts, with 29 participants raising at least one alert; 88 (53.5%) alerts received a call discussing self-management, of which 35 (21%) received definitive advice that may otherwise have required an unscheduled healthcare visit. There was a greater reduction in median exacerbation rate across both telehealth groups at 6 months post-intervention (1 to 0, *p* < 0.001) but not in control groups (0.5 to 0.0, *p* = 0.121). Similarly, there was a significant reduction in unscheduled GP visits across the telehealth groups (1.5 to 0.0, *p* < 0.001), but not the control groups (0.5 to 0.0, *p* = 0.115). These reductions led to cost-savings across all groups, but greater in the telehealth cohorts. The addition of telehealth support to exacerbation-prone patients with asthma or COPD, following comprehensive assessment and treatment optimisation, proved beneficial in reducing exacerbation frequency and unscheduled healthcare visits and thus leads to significant cost-savings for the NHS.

Clinical Trial Registration: ClinicalTrials.gov: NCT03096509

## Introduction

Across the United Kingdom (UK), nearly 1 in 5 people are diagnosed with a respiratory condition during their lifetime, with asthma and chronic obstructive pulmonary disease (COPD) accounting for approximately half of all new respiratory diagnoses^1^. The personal and economic cost of poor disease control is high. Annually, it is estimated that asthma and COPD cost the National Health Service (NHS) £3 billion and £1.9 billion retrospectively, partly driven by a significant increase in hospital admissions over the recent years^2,3^.

The NHS Long Term Plan aims to integrate respiratory services around the patient; to detect and diagnose respiratory conditions at an earlier stage and enable patients to manage their own health in the community, supplemented by expert advice and peer support^4^. It is well established that supporting self-management can improve disease control and reduce unscheduled care visits in both asthma and COPD^5–8^. Technology is increasingly integrated into every aspect of life and it is reasonable to assume digital systems may be used to help support the self-management of long-term conditions and in particular respiratory diseases.

With recent advances in technology, telehealth services and/or remote monitoring systems are being utilised with increasing frequency and have been proven to be effective in helping patients manage chronic conditions, including type 1 diabetes and hypertension^9,10^. Notably, the use of telehealth services has grown exponentially throughout the current COVID-19 era^11,12^ with patients being reluctant to attend General Practices (GP’s) or hospital clinics for appointments. However, it is important to ensure there is ongoing healthcare interaction for these patients with chronic conditions and the use of telehealth services provided a means for this. Alternative methods promoting the use of a self-management plan for patients with asthma or COPD could also have a beneficial financial impact given the effect of reduced exacerbations or admissions to hospital.

We hypothesised that the addition of telehealth support could lead to a reduction in unscheduled care during delivery of an integrated respiratory clinic in patients with asthma, COPD and suspected respiratory causes of breathlessness.

## Methods

### Multi-disciplinary team clinics

MISSION-ABC (Modern innovative solutions to improve outcomes in asthma, breathlessness, and chronic obstructive pulmonary disease) was an observational study sponsored by Portsmouth Hospitals University NHS Trust evaluating the impact of delivering multi-disciplinary respiratory clinics led by integrated primary and secondary care teams and delivered largely in primary care practices^13^. It was approved by the South-Central Berkshire Research Ethics Committee (Reference 16/SC/0646) and registered at ClinicalTrials.gov, registration number NCT03096509^14^. Further details of this study are publicly available at https://missionabc.uk.

Fifty MISSION-ABC clinics were held between September 2016 and July 2017, with 441 participants recruited with written informed consent from eleven GP surgeries across South-East Hampshire. Patients aged ≥16 years with poor asthma or COPD control (identified by frequent exacerbations, emergency department visits, hospital admissions, use of three or more controller medications or frequent use of short-acting bronchodilators), a high symptom burden from their asthma or COPD, or those with undifferentiated breathlessness were invited to attend a MISSION-ABC clinic. This clinic consisted of a comprehensive multi-disciplinary team (MDT) assessment with the team including secondary care respiratory nurse specialists, respiratory physiotherapist, respiratory physiologist and respiratory doctors together with healthcare practitioners from the primary care practice. Each participant underwent assessments including spirometry (MicroLab 3500, CareFusion, UK), oscillometry (Tremoflo^®^, Thorasys, CA), measurement of fractional exhaled nitric oxide (FeNO) (NIOX Mino^®^ Circassia, UK), disease control and comorbidity questionnaires, breathing pattern and inhaler technique assessments alongside a specialist medical review. Reasons behind poor disease control including comorbidities, difficulties in self-management and social stressors were addressed and medications were optimised in line with local and national guidelines. A personalised self-management (or action) plan with inhaler technique information was written with participants before they left the clinic.

The outcomes of the participants assessments by all members of the MDT were collated and discussed, with those with poor disease control and considered at risk of exacerbations identified as eligible for the e-platform monitoring solution. Criteria for the e-platform included evidence of uncontrolled disease, with access to a telephone and a willingness to; use the telephone platform, respond to clinical team alerts, follow clinical advice and potentially travel to the hospital for further assessment if required. This allowed remote monitoring of symptoms for up to three months with telephone support provided if required. The e-platform technology was provided by Monitor^®^ (Message Dynamics^®^, Chertsey UK, www.messagedynamics.co.uk).

### Telehealth alerts

Participants received an automated telephone call twice a week on pre-determined days. The automated call was from a script which varied depending upon the participants underlying respiratory condition, and included a series of questions to assess their current symptom burden, for example:

- How short of breath are you today compared to when we last called you?
- How wheezy is your chest since our last call?
- Do you have a new cough since our last call?
- Has your asthma been waking you up at night since our last call?
- Have you been using your reliever inhaler more than usual?

Each response was linked to an alert sent to the specialist respiratory research team by email and also to a dedicated web portal which the team could monitor. The research team logged on at least twice a day (Monday-Friday) to respond to any new alerts. Participants who self-reported at least one symptom or an increase in symptom burden were contacted by the research team, with specialist respiratory nurse advice offered over the telephone where required. Any further concerns or triggers were escalated to a duty respiratory research physician for review.

Supportive telephone calls included reminders about using their inhalers as maintenance and reliever therapy (MART) and encouragement to follow their self-management plan. Frequent interventions included medication advice (e.g. increase their inhaler usage, start oral corticosteroids and/or antibiotics), an outpatient respiratory appointment or they were advised to see their GP.

### Follow up

Participant data were collected at baseline which included information about the previous 6 months, with questionnaires completed before or at the first MISSION-ABC clinic and then at 3 and 6 months. Healthcare utilisation (e.g. prescriptions, unscheduled GP visits, emergency department (ED) visits and hospital admissions) was collected from electronic medical records at 6- and 12-months post MISSION-ABC clinic. Unscheduled GP visits were defined as a non-elective visit to the GP practice within hours, with out-of-hours GP attendance defined as a non-elective visit outside of standard working hours. An exacerbation was defined as an acute flare of their lung disease requiring a course of oral corticosteroids and/or antibiotics. Telehealth support was used for 3 months following their MISSION-ABC clinic and data was securely managed using Microsoft^®^ Access^®^ (Microsoft Corp., Redmond, WA, USA).

### Analyses

A matched cohort study was performed to assess the additional benefit that a telehealth service offered following a comprehensive assessment with treatment optimisation as part of the MISSION-ABC clinic.

Participants were sought to be matched by factors likely to affect the study outcome:

- Post MISSION-ABC diagnosis (Asthma or COPD as diagnosed by a secondary care physician following national guidelines and agreed at a respiratory MDT)
- Age (±10 years)
- Sex
- FEV_1_ percent predicted (forced expiratory volume in one second) (within 10%)
- Smoking status (current, ex-smoker, or never smoked)
- A minimum of 1 exacerbation in the previous twelve months

Unscheduled care use, including exacerbations and hospital admissions, and questionnaire responses between the telehealth and control cohorts were compared using Wilcoxon tests (two-sided) for nonparametric paired analyses as appropriate for a matched cohort study.

### Cost-effectiveness

It was anticipated that following treatment optimisation in a multi-disciplinary clinic there would be a reduction in exacerbation frequency and unscheduled GP visits; therefore determining the additional benefit of a telehealth service was performed as a matched cohort study. The cost-effective analyses included information on unscheduled care visits in primary care, emergency department visits, hospital admissions, the cost of the tele-health system per participant and the clinical time required for twice weekly triage and alert handling by clinical teams. Standard NHS templates were used to construct the total costs^15,16^. The analyses compared the cost 6 months pre- and post-initiating the telehealth intervention. The analyses were stratified by cases and controls, and by the post clinic diagnosis of either asthma or COPD.

### Reporting summary

Further information on research design is available in the Nature Research Reporting Summary linked to this article.

## Results

**Fig. 1:**
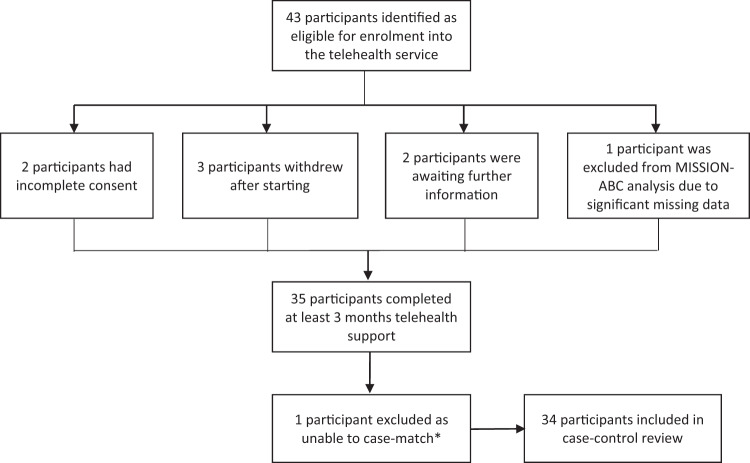
Flow-chart demonstrating participant recruitment. Following a review in a MISSION-ABC clinic, one participant did not meet the inclusion criteria of a confirmed diagnosis of asthma or COPD.

Forty-three participants were identified as eligible for enrolment into the telehealth service (Fig. 1). Two participants had incomplete consent and two were awaiting further information before enrolment started. Three participants withdrew after starting, and one participant was not included in the final MISSION-ABC analysis due to significant missing data. Thirty-five participants completed at least 3 months telehealth support following assessment and treatment optimisation in a MISSION-ABC clinic and were included in the analyses.

The 34 telehealth participants with a MISSION-ABC confirmed diagnosis of asthma or COPD were matched with 29 participants in the control arm; all of whom had at least one exacerbation in the previous 12 months. Participant demographics are shown in Table 1; 17 participants in the telehealth cohort had a diagnosis of asthma, with 17 a diagnosis of COPD matched to 12 and 16 participants in the control cohorts respectively. FeNO was higher in the asthma telehealth cohort, consistent with a population more likely to experience recurrent exacerbations. Having more than one comorbidity was not intentionally matched but is similar between the telehealth and control cohorts. The average (median) body mass index (BMI) was also similar between the telehealth and control groups within disease categories (30.1 in telehealth and 30.4 in the control group within the asthma cohort, and within the COPD cohort 24.9 in telehealth and 26.4 in the control group). Most of our a priori matching criteria was met except for sex in the asthma telehealth group where there were 7 out of 17 males in telehealth compared to only 1 out of 12 in the control group. We included this person in our control arm as they otherwise met the matching criteria.

**Table 1:** Participant characteristics.

Cohort	Asthma		COPD		Total	
	Telehealth	Control	Telehealth	Control	Telehealth	Control
***N***	17	12	17	16	34	28
**Demographics**						
Age (years)	62 [43,68]	64 [56.3, 70.3]	65 [59,70]	69.5 [66, 72.5]	64 [57,70]	68.5 [61.8, 72]
Male (%)	7 (41.2)	1 (8.33)	8 (47.1)	8 (50.0)	15 (44.1)	9 (32.1)
BMI kg/m^2^	30.1 [27.3, 33.6]	30.4 [25.2, 33.5]	24.9 [22.6. 31.7]	26.4 [23.4, 30.1]	29.4 [24.5, 33.6]	27.8 [24.6, 32.6]
**Lung function**						
FEV_1_ % predicted	70.4 ± 22.8	79.5 ± 17.4	54.4 ± 16.0	43.1 ± 22.3	62.1 ± 21.1	58.5 ± 27.2
FeNO ppb	17 [11.5, 45]	16 [9, 30.5]	9 [6,12]	18.5 [12, 27.8]	12 [9,25]	16 [9.5, 28.5]
**Smoking status**						
Current smoker (%)	5 (29.4)	5 (41.7)	10 (58.8)	6 (37.5)	15 (44.1)	11 (39.3)
Ex-smoker (%)	9 (52.9)	6 (50.0)	7 (41.2)	10 (62.5)	16 (47.1)	16 (57.1)
Never smoker (%)	3 (17.6)	1 (8.3)	0	0	3 (8.8)	1 (3.6)
**Comorbidities**						
≥1 (%)	10 (58.8)	7 (58.3)	14 (82.4)	15 (93.8)	24 (70.6)	22 (78.6)
Cardiovascular (%)	7 (41.2)	5 (41.7)	5 (29.4)	9 (56.3)	12 (35.3)	14 (50)
Gastrointestinal (%)	6 (35.3)	2 (16.7)	5 (29.4)	6 (37.5)	11 (32.4)	8 (28.6)
Diabetes (%)	6 (35.3)	1 (8.3)	4 (23.5)	5 (31.3)	10 (29.4)	6 (21.4)

Values are median [Q1, Q3], number (%) or mean ± SD

*BMI* body mass index, *FEV*_*1*_ forced expiratory volume in one second, *FeNO* Fraction of exhaled nitric oxide.

We compared demographics, lung function, clinical outcomes and questionnaire responses in participants included in the telehealth group versus all those not included, stratified by disease category (see supplementary information). This confirms participants in the telehealth asthma cohort had lower lung function; participants in the telehealth COPD cohort had lower FeNO, more ex-smokers and were younger; with both telehealth asthma and COPD cohorts having a higher rate of exacerbations as would be expected based on our selection criteria. There were some minor statistically significant differences in co-morbidities.

Across both asthma and COPD telehealth cohorts, there were 165 triggers via the electronic alert system. 72 triggers (43.6%) were for participants diagnosed with asthma (mean 4.2 triggers per participant) and 93 (56.4%) triggers from participants with a COPD diagnosis (mean 5.5 triggers per participant). The alerts were triggered by 29 of the 34 telehealth participants, with 16/17 (94%) participants with COPD and 13/17 (76%) participants with asthma raising at least one alert.

Of those 165 alerts, 88 (53.5%) required a supportive call with discussion regarding self-management, 37 (22.4%) did not respond to subsequent telephone calls, 3 (1.8%) had reviews already planned on the day of triggering so no further action was needed and 2 (1.2%) were triggers in error (Table 2).

**Table 2:** Telehealth alerts.

	Supportive call	Advice or action	No answer	Planned review	Triggered in error
Asthma (%)	35 (39.8)	19 (54.3)	15 (40.5)	2 (66.6)	1 (50)
COPD (%)	53 (60.2)	16 (45.7)	22 (59.5)	1 (33.3)	1 (50)
Total	88	35	37	3	2

Values are number (%).

### Telehealth triggers and participants

A total of 35 triggers (21.2%) were managed with definitive advice or action that otherwise may have required attendance at the patients’ GP surgery. For these 35 triggers: outpatient clinic appointments were arranged for 9, with a further participant discussed in the Portsmouth Hospital severe asthma service MDT; 17 triggers were answered with advice regarding inhalers or other medications including antibiotics and oral corticosteroids; 3 participants were advised to see their GP; 1 was given sputum clearance advice; 1 participant was highlighted for a medical review but declined to speak to a doctor and 3 participants were escalated to a doctor for further intervention given the deterioration in their symptoms. These participants were not brought back to a clinic or admitted, but the outcome following those conversations is unclear.

### Unscheduled care use

Table 3 demonstrates the number of exacerbations and healthcare visits, comparing the 6 months prior to MISSION-ABC clinic to 6 months post MISSION-ABC clinic in each cohort. In the 6 months prior to the MISSION-ABC clinic, there were a total of 23 exacerbations in the asthma telehealth cohort, improving to 9 exacerbations across the group in the 6 months following the MISSION-ABC clinic (*p* = 0.006). The number of unscheduled GP visits fell from 54 urgent appointments prior to the MISSION-ABC clinic to 14 in the 6 months post clinic (*p* = 0.008). Likewise, in the COPD telehealth cohort, there were 37 exacerbations in the 6 months prior to the MISSION-ABC clinic, improving to 18 exacerbations in the 6 months post MISSION-ABC (*p* = 0.017), with a similar reduction in unscheduled GP visits from 39 pre clinic to 13 post MISSION-ABC (*p* = 0.005). In our control cohorts, both exacerbation frequency and unscheduled GP visits reduced, but no reduction was statistically significant. Exacerbation frequency reduced from 6 to 3 in our asthma control cohort (*p* = 0.317), and from 21 to 14 in our COPD control cohort (*p* = 0.223), with unscheduled GP visits falling from 9 to 4 visits in the asthma controls (*p* = 0.301) and from 28 to 17 in the COPD control (*p* = 0.215).

**Table 3:** Paired samples showing exacerbations, out-of-hours, and unscheduled healthcare visits.

	Cohort	Asthma						COPD						Total					
	Timepoint	Pre			Post			Pre			Post			Pre			Post		
Health service		*N*	Total events	Average	Total events	Average	*p* value	*N*	Total events	Average	Total events	Average	*p* value	*N*	Total events	Average	Total events	Average	*p* value
**Exacerbations**	**Telehealth**	16	23	1.0 [0.0, 2.2]	9	0.0 [0.0, 1.0]	0.006	17	37	1.0 [1.0, 4.0]	18	0.0 [0.0, 1.0]	0.017	33	60	1.0 [1.0, 3.0]	27	0.0 [0.0, 1.0]	<0.001
	**Control**	12	6	0.0 [0.0, 1.0]	3	0.0 [0.0, 0.2]	0.317	16	21	1.0 [0.0, 3.0]	14	0.0 [0.0, 1.2]	0.223	28	27	0.5 [0.0, 1.2]	17	0.0 [0.0, 1.0]	0.121
**OOH attendance**	**Telehealth**	17	3	0 [0, 0]	2	0 [0, 0]	0.705	17	3	0 [0, 0]	0	-	-	34	6	0 [0, 0]	2	0 [0, 0]	0.336
	**Control**	12	0	-	0	-	-	16	4	0 [0, 0]	0	-	-	28	4	0 [0, 0]	0	-	-
**ED attendance**	**Telehealth**	17	3	0 [0, 0]	2	0 [0, 0]	0.785	17	3	0 [0, 0]	0	-	-	34	6	0 [0, 0]	2	0 [0, 0]	0.357
	**Control**	12	0	-	0	-	-	16	0	-	0	-	-	28	0	-	0	-	-
**Hospital admissions**	**Telehealth**	17	0	-	0	-	-	17	3	0 [0, 0]	0	-	-	34	3	0 [0, 0]	0	-	-
	**Control**	12	0	-	0	-	-	16	1	0 [0, 0]	0	-	-	28	1	0 [0, 0]	0	-	-
**Unscheduled GP visits**	**Telehealth**	17	54	2.0 [0.0, 4.0]	14	0.0 [0.0, 2.0]	0.008	17	39	1.0 [0.0, 4.0]	13	0.0 [0.0, 1.0]	0.005	34	93	1.5 [0.0, 4.0]	27	0.0 [0.0, 1.0]	<0.001
	**Control**	12	9	0.0 [0.0, 1.0]	4	0.0 [0.0, 0.2]	0.301	16	28	1.0 [0.0, 3.2]	17	0.0 [0.0, 1.2]	0.215	28	37	0.5 [0.0, 3.0]	21	0.0 [0.0, 1.0]	0.115
**ACQ**	**Telehealth**	5	14.2	2.6 [2.3, 4.0]	9.7	1.8 [1.5, 2.0]	0.279												
	**Control**	3	4.6	1.4 [1.4, 1.6]	4.5	1.7 [1.2, 1.8]	1												
**CAT**	**Telehealth**							1	22	22.0 [22.0, 22.0]	20	20.0 [20.0, 20.0]	-						
	**Control**							5	94	15.0 [14.0, 19.0]	125	23.0 [23.0, 28.0]	0.042						
**ASK**	**Telehealth**	2	43	21.5 [20.8, 22.2]	49	24.5 [23.2, 25.8]	0.18	0	0	-	0	-	-	2	43	21.5 [20.8, 22.2]	49	24.5 [23.2, 25.8]	0.18
	**Control**	4	69	17.0 [15.5, 18.8]	71	17.5 [14.8, 20.5]	1	5	94	18.0 [17.0, 21.0]	95	17.0 [17.0, 22.0]	1	9	163	18.0 [16.0, 21.0]	166	17.0 [15.0, 22.0]	0.943
**PAM**	**Telehealth**	5	310.3	51.0 [51.0, 63.1]	294.0	55.6 [51.0, 55.6]	0.684	2	106.6	53.3 [52.1, 54.5]	93.11	46.6 [44.4, 48.8]	0.18	7	416.8	51.0 [51.0, 59.3]	387.1	51.0 [49.0, 55.6]	0.307
	**Control**	4	250.7	63.0 [55.3, 70.3]	244.9	61.7 [53.9, 69.0]	0.285	6	377.8	67.8 [55.2, 71.4]	339.32	53.2 [53.2, 64.2]	0.046	10	628.5	67.8 [52.7, 71.4]	584.2	54.4 [53.2, 67.8]	0.021

Median [Q1, Q3]

*OOH* Out of hours, *ED* emergency department, *GP* general practitioner, *ACQ* asthma control questionnaire, *CAT* COPD assessment test, *ASK* adherence starts with knowledge, *PAM* patient activation measure

### Questionnaires

The ACQ (Asthma Control Questionnaire) and CAT (COPD Assessment Tool) scores were used to assess patient reported level of disease control on the day of their MISSION-ABC clinic, and then 6 months later. ACQ scores improved in the telehealth cohort, although this was not statistically significant. The number of patients completing a CAT score was too small to allow meaningful comparison. Medication adherence was assessed using the ASK-12 (Adherence Starts with Knowledge) questionnaire with no statistical difference identified. The Patient Activation Measure (PAM) questionnaire was also compared, with a small but significantly reduced activation in the control COPD cohort (in a group of 6 participants).

### Cost-effectiveness

Table 4 shows the costs of unscheduled care between telehealth and controls comparing cost pre and post MISSION-ABC clinic. The reduction in exacerbations, unscheduled GP appointments and admissions resulted in overall cost savings across all groups. However, this was greater in the telehealth cohort. Per exacerbation, there was a direct saving of £1.74 and £4.59 in the control and telehealth cohorts respectively. For hospital admissions, there were direct cost savings of £205.69 and £580.76 per participant in the control and telehealth cohorts respectively. There was a reduction in overall costs for the telehealth intervention across all five measures of unscheduled care use. The largest savings were seen within hospital admissions and unscheduled GP visits. The asthma groups showed larger reductions in costs associated with unscheduled GP visits than COPD. Conversely, the COPD cohort showed substantial reductions in hospital admission costs. The cost reductions across other measures of unscheduled care were broadly similar. The cost of the telehealth intervention averaged £12 per participant for the three months of the study. Overall, the addition of telehealth proved a cost-effective measure, saving an average £444.35 per participant.

**Table 4:** Mean cost per participant pre and post MISSION-ABC clinic for unscheduled care use between telehealth and controls.

		Asthma			COPD			All			Cost difference
		Pre	Post	Diff	Pre	Post	Diff	Pre	Post	Diff	
Exacerbations	Telehealth	£6.60	£2.87	−£3.73	£10.62	£5.17	−£5.45	£8.61	£4.02	−£4.59	−£2.85
	Control	£2.44	£1.22	−£1.22	£6.41	£4.27	−£2.14	£4.71	£2.96	−£1.74	
Out of hours GP	Telehealth	£12.33	£8.22	−£4.11	£12.33	£0.00	−£12.33	£12.33	£4.11	−£8.22	£1.76
	Control	£0.00	£0.00	£0.00	£17.47	£0.00	−£17.47	£9.98	£0.00	−£9.98	
Unscheduled GP	Telehealth	£140.11	£36.33	−£103.79	£101.19	£33.73	−£67.46	£120.65	£35.03	−£85.63	−£60.42
	Control	£33.08	£14.70	−£18.38	£77.19	£46.87	−£30.33	£58.29	£33.08	−£25.21	
ED Attendance	Telehealth	£29.65	£19.76	−£9.88	£29.65	£0.00	−£29.65	£29.65	£9.88	−£19.76	−£19.76
	Control	£0.00	£0.00	£0.00	£0.00	£0.00	£0.00	£0.00	£0.00	£0.00	
Hospital Admission	Telehealth	£0.00	£0.00	£0.00	£580.76	£0.00	−£580.76	£580.76	£0.00	−£580.76	−£375.08
	Control	£0.00	£0.00	£0.00	£205.69	£0.00	−£205.69	£205.69	£0.00	−£205.69	
All unscheduled	Telehealth	£188.70	£67.18	−£121.51	£734.56	£38.90	−£695.66	£752.01	£53.04	−£698.97	−£456.35
	Control	£35.52	£15.92	−£19.60	£306.76	£51.14	−£255.62	£278.66	£36.05	−£242.62	
							**Cost of Telehealth per participant:**				**£12.00**
							**Total Savings per participant:**				**£444.35**

## Discussion

The addition of telehealth support to an exacerbation-prone population, following assessment and treatment optimisation in a MISSION-ABC clinic, proved beneficial in reducing both the frequency of exacerbations and unscheduled healthcare visits. These reductions also proved to be cost effective. This is the first report of an integrated respiratory clinic delivered in primary care that has shown that the use of telehealth using telephone triggers led to a combined reduction in unscheduled care use. Here we discuss the results of our study and the limitations.

The MISSION-ABC clinic provided patients with a multi-disciplinary assessment, including treatment optimisation, education and the development of a self-management plan. It was therefore anticipated there would be an improvement in exacerbation frequency and unscheduled GP visits, hence determining the additional benefit of a telehealth service was performed as a matched cohort study. As this is a post-hoc analysis, the control and telehealth cohorts are not identical in size, however the participants are matched by their post MISSION-ABC diagnosis alongside factors that may influence their disease trajectory and unscheduled care use including age, sex, smoking history and FEV_1_ percent predicted.

MISSION-ABC encompassed participatory action research (PAR) methodology to evolve the clinic interventions. This therefore raises the possibility of a population bias as participants who engage with PAR may also be more likely to engage with a telehealth service. However, both our telehealth and control cohorts had attended the MISSION-ABC clinics and were therefore likely to be similarly engaged. One advantage of MISSION-ABC is that clinics were held in multiple GP practices in South East Hampshire allowing inclusion of participants across the region from different socio-economic areas which contributes to the generalisation of results.

Comparisons were performed between 6 months pre- and post- the MISSION-ABC clinic as per study protocol. We recognise that seasonal trends could affect the outcomes for an analysis period that encompasses only 6 months follow-up for an intervention that only lasted 3 months. We believe this bias has been minimised by recruiting into MISSION-ABC over an 11-month period thereby reducing any overall impact of secular trends.

As expected following the MISSION-ABC clinic, exacerbation frequency and unscheduled GP visits improved across all groups. There was a statistical difference in the reduction of exacerbations across all participants who engaged with the telehealth service, although this is less pronounced when subdivided by disease category. Importantly, there was a significant reduction in the number of unscheduled GP visits for participants in the telehealth cohorts with both asthma and COPD. The addition of the telehealth service provided the reassurance and encouragement needed for participants to follow their self-management plan at home, whether that was to increase their inhaled corticosteroid, or to start a course of steroids and/or antibiotics.

The attrition rate for returning questionnaires was high and not consistent across the groups making data from smaller samples difficult to interpret. For example, there was an improvement in the ACQ score in the telehealth cohort, although this did not reach statistical significance. Similarly there was a reduction in the PAM score in the COPD control group. These observations would require more detailed exploration in future studies.

For just over 50% of the alerts received, a supportive phone call by a trained healthcare professional with discussion of their self-management plan was sufficient. This supportive phone call and encouragement to follow their self-management plan was provided in a timely manner, pro-actively recognising and acting upon a change in symptoms. This early detection of deterioration allowed timely intervention to prevent any further decline which may have resulted in a more severe exacerbation, unscheduled GP visit or even hospital admission. Twenty-one percent of the triggers resulted in definitive advice or action being provided, with the majority relating to medications including the need to start antibiotics or oral corticosteroids. Of the 165 triggers, a clinical review was suggested for only 12 participants (7%), indicating that most alerts can be successfully managed with remote support. Despite triggering on a telehealth service designed to recognise an increased symptom burden, 22% of participants did not respond to a subsequent telephone call. As a telehealth service requires regular engagement from the participant, there will naturally be a bias towards those who will use and subsequently benefit from a remote service. We have also considered the bias from not being able to contact participants after alerts. The study methodology did not allow us to interrogate for reasons why participants were unable to be contacted. Nevertheless, their data was included in the analysis. Given that this was a substantial proportion of alerts, future research needs to explore reasons why participants could not be reached as this is a missed opportunity for an intervention. Conceivably, our reduction in unscheduled healthcare use may have been greater had we been able to contact these participants. Following a telehealth alert, all participants received a telephone call from a specialist nurse. Some calls went unanswered and although we accept the resource implications of this, this was not included in our cost-effective analysis recognising the time required for this was at most a few minutes and no subsequent interventions were required.

We compared the telehealth group versus all other participants and predictably they had lower lung function, higher rate of exacerbations and a higher FeNO as would have been expected based on our selection criteria. This supports the rationale that our intervention is more likely to be of benefit in those with more severe disease.

Overall, the cost of the telehealth intervention per participant was low (£12) for the three months of the study. This cost compares favourably to the average cost of an inhaler for three months. This intervention led to significant reductions in unscheduled care use over and above any benefit realised from simply attending a MISSION-ABC clinic i.e. our controls. Overall, the addition of telehealth proved a cost-effective measure, saving an average £444.35 per participant, and a large part of reduction in costs was in hospital admissions and unscheduled GP visits. We accept that the cost may have been underestimated as we were unable to contact a proportion of participants triggering an alert.

The potential advantage of telehealth in respiratory disease has been recognised but its value remains unproven. In 2011, a systematic review suggested telehealth may improve quality of life and reduce the number of hospital and ED visits in COPD^17^. A more recently published systematic review regarding digital interventions in managing COPD further concluded that there was no evidence of harm from digital interventions, but also no clear evidence of long-term benefit either^18^. It is a similar story for patients with asthma; a 2016 Cochrane review of home telemonitoring for patients with asthma also concluded there was no clear evidence of benefit, or harm from this intervention^19^. We have, however, shown a benefit in asthma and COPD with evidence of cost effectiveness, although acknowledge this is in a small cohort.

This improvement may be explained as participants were enroled from an exacerbation-prone population, following thorough assessment and treatment optimisation in a MISSION-ABC clinic. The addition of telehealth provided additional personal and interactive support in self-management and reduced the reliance on a GP appointment. This telehealth support does not replace GP or clinical visits, and at times, participants were actively encouraged to seek GP advice, however, the addition of telehealth does empower participants to self-manage at home where appropriate. Digital health is a developing market, with an ever-increasing number of technological interventions available. Using these to support self-management at home will be key in the years to come, however identifying appropriate patients is also a crucial component. This matched cohort review shows that telehealth support can reduce the number of unscheduled GP appointments in patients with both asthma and COPD, but a large-scale randomised control trial is required to prove long-term benefit.

In conclusion, the development of a self-management plan and the use of telehealth support following treatment optimisation provides an opportunity to detect early signs of deterioration, to reassure and to encourage the use of self-management plans with a subsequent reduction in the frequency of exacerbations and unscheduled GP visits. In a post COVID-19 era, where there will be an increasing focus on the use of remote technology, this study supports the hypothesis that telehealth services can be key in chronic disease management for patients with asthma and COPD.

## Supplementary information

Supplementary Information

REPORTING SUMMARY

## Data Availability

The clinical trial data, including third-party information, can be made available upon request via email to the corresponding author and a link to a secure file will be shared.
