# Supplementary material for: Evaluation of telehealth support in an integrated respiratory clinic
Source: NPJ Prim Care Respir Med. 2022 Nov 11;32:51. doi: 10.1038/s41533-022-00304-9 (PMC9650174; doi:10.1038/s41533-022-00304-9)
Supplement: Supplementary file 1 — Supplementary Information [file 41533_2022_304_MOESM1_ESM.pdf]

Supplementary Information: Table of all participants with a post-clinic diagnosis of asthma or COPD in MISSION-ABC.

	Asthma			COPD			Total		
	Telehealth	Standard Care	P-value	Telehealth	Standard Care	P-value	Telehealth	Standard Care	P-value
N	17	227		17	121		34	348	
**Demographics**									
Age	62.0 [43.0, 68.0]	58.0 [48.0, 71.0]	0.438	65.0 [59.0, 70.0]	73.0 [66.0, 79.0]	0.003	64.0 [57.0, 70.0]	66.0 [52.0, 74.0]	0.182
Male	7 (41.2%)	67 (29.5%)	0.462	8 (47.1%)	66 (54.5%)	0.749	15 (44.1%)	133 (38.2%)	<0.001
BMI	30.1 [27.3, 33.6]	28.1 [25.0, 32.9]	0.106	24.9 [22.6, 31.7]	26.6 [23.3, 30.7]	0.337	29.4 [24.5, 33.6]	27.6 [24.4, 32.4]	0.266
**Lung function**									
FeNO	17.0 [11.5, 45.0]	21.0 [13.0, 33.0]	0.489	9.0 [6.0, 12.0]	17.0 [11.0, 26.0]	0.001	12.0 [9.0, 25.0]	20.0 [12.0, 30.0]	0.017
FEV_1 % predicted	70.4 ± 22.8	86.6 ± 20.1	0.004	54.4 ± 16.0	57.2 ± 21.2	0.623	62.1 ± 21.1	76.7 ± 24.8	0.002
**Smoking status**									
Current	5 (29.4%)	41 (18.1%)		10 (58.8%)	31 (25.8%)		15 (44.1%)	72 (20.8%)	
Ex	9 (52.9%)	79 (35.0%)	0.134	7 (41.2%)	87 (72.5%)	0.046	16 (47.1%)	166 (48.0%)	<0.001
Never	3 (17.6%)	106 (46.9%)		-	2 (1.7%)		3 (8.8%)	108 (31.2%)	
**Comorbidities**									
Cardiovascular	7 (41.2%)	82 (36.1%)	0.876	5 (29.4%)	61 (50.4%)	0.173	12 (35.3%)	143 (41.1%)	0.055
Diabetes	6 (35.3%)	24 (10.6%)	0.009	4 (23.5%)	23 (19.0%)	0.910	10 (29.4%)	47 (13.5%)	0.009
Gastrointestinal	6 (35.3%)	72 (31.7%)	0.972	5 (29.4%)	27 (22.3%)	0.732	11 (32.4%)	99 (28.4%)	0.286
>1 comorbidity	10 (58.8%)	167 (73.6%)	0.302	14 (82.4%)	91 (75.2%)	0.731	24 (70.6%)	258 (74.1%)	0.432
**Exacerbations**									
Pre-clinic exacerbations	1.4 ± 1.3	0.7 ± 0.9	0.011	2.2 ± 2.0	1.2 ± 1.3	0.007	1.8 ± 1.7	0.9 ± 1.1	<0.001
Post-clinic exacerbations	0.6 ± 0.8	0.3 ± 0.6	0.047	1.1 ± 1.8	0.6 ± 1.0	0.148	0.8 ± 1.4	0.4 ± 0.8	0.007
>1 exacerbations	11 (64.7%)	112 (49.3%)	0.332	14 (82.4%)	74 (61.2%)	0.152	25 (73.5%)	186 (53.4%)	0.014
**Questionnaires**									
ASK	25.5 ± 3.8	21.4 ± 5.4	0.069	-	22.0 ± 6.1	-	25.5 ± 3.8	21.6 ± 5.6	0.093
PAM	59.5 ± 17.2	62.0 ± 13.3	0.644	58.1 ± 7.1	55.9 ± 8.6	0.664	59.1 ± 15.0	59.9 ± 12.3	0.838
WPAI	4.7 ± 3.0	2.4 ± 2.5	0.018	8.0 ± 0.0	4.0 ± 2.8	0.048	5.4 ± 3.0	2.9 ± 2.7	0.006

Values presented as:
Median [Q1, Q3] compared using independent t-tests
Number (%) compared using chi square contingency
Mean ± SD compared using Mann-Whitney test
