# Supplementary material for: Evaluation of telehealth support in an integrated respiratory clinic
Source: NPJ Prim Care Respir Med. 2022 Nov 11;32:51. doi: 10.1038/s41533-022-00304-9 (PMC9650174; doi:10.1038/s41533-022-00304-9)
Supplement: Supplementary file 2 — REPORTING SUMMARY [file 41533_2022_304_MOESM2_ESM.pdf]

nature portfolio

Corresponding author(s): Lauren Fox

Last updated by author(s): Aug 8, 2022

Reporting Summary

Nature Portfolio wishes to improve the reproducibility of the work that we publish. This form provides structure for consistency and transparency in reporting. For further information on Nature Portfolio policies, see our Editorial Policies and the Editorial Policy Checklist.

Statistics

For all statistical analyses, confirm that the following items are present in the figure legend, table legend, main text, or Methods section.

n/a	Confirmed	
☐	☒	The exact sample size (n) for each experimental group/condition, given as a discrete number and unit of measurement
☐	☒	A statement on whether measurements were taken from distinct samples or whether the same sample was measured repeatedly
☐	☒	The statistical test(s) used AND whether they are one- or two-sided<br>*Only common tests should be described solely by name; describe more complex techniques in the Methods section.*
☐	☒	A description of all covariates tested
☐	☒	A description of any assumptions or corrections, such as tests of normality and adjustment for multiple comparisons
☐	☒	A full description of the statistical parameters including central tendency (e.g. means) or other basic estimates (e.g. regression coefficient) AND variation (e.g. standard deviation) or associated estimates of uncertainty (e.g. confidence intervals)
☐	☒	For null hypothesis testing, the test statistic (e.g. F, t, r) with confidence intervals, effect sizes, degrees of freedom and P value noted<br>*Give P values as exact values whenever suitable.*
☒	☐	For Bayesian analysis, information on the choice of priors and Markov chain Monte Carlo settings
☒	☐	For hierarchical and complex designs, identification of the appropriate level for tests and full reporting of outcomes
☒	☐	Estimates of effect sizes (e.g. Cohen's d, Pearson's r), indicating how they were calculated

Our web collection on statistics for biologists contains articles on many of the points above.

Software and code

Policy information about availability of computer code

Data collection	Data was collected, cleaned and secured using the REDCap secure cloud system.
Data analysis	Data was analysed using both Excel and SPSS.

For manuscripts utilizing custom algorithms or software that are central to the research but not yet described in published literature, software must be made available to editors and reviewers. We strongly encourage code deposition in a community repository (e.g. GitHub). See the Nature Portfolio guidelines for submitting code & software for further information.

Data

Policy information about availability of data

All manuscripts must include a data availability statement. This statement should provide the following information, where applicable:

- Accession codes, unique identifiers, or web links for publicly available datasets
- A description of any restrictions on data availability
- For clinical datasets or third party data, please ensure that the statement adheres to our policy

The clinical data sets includes third party data. This along with the clinical trial data will be made available upon request by email to the leading author. Access will be provided by a file share link. The files will be in an executable excel file.

Human research participants

Policy information about studies involving human research participants and Sex and Gender in Research.

Reporting on sex and gender	We reported sex throughout the study.
Population characteristics	The index population were participants receiving a telehealth intervention compared to age and sex matched controls who did not receive this additional telehealth intervention. All participants were recruited from primary care community respiratory clinics, and were further stratified by their confirmed diagnosis of either asthma or COPD.
Recruitment	Recruitment was part of a regulated clinical research study within the national health service setting. All participants were recruited from primary care community respiratory clinics.
Ethics oversight	The research study was approved by the South-Central Berkshire Research Ethics Committee (Reference 16/SC/0646).

Note that full information on the approval of the study protocol must also be provided in the manuscript.

Field-specific reporting

Please select the one below that is the best fit for your research. If you are not sure, read the appropriate sections before making your selection.

☒ Life sciences ☐ Behavioural & social sciences ☐ Ecological, evolutionary & environmental sciences

For a reference copy of the document with all sections, see nature.com/documents/nr-reporting-summary-flat.pdf

Life sciences study design

All studies must disclose on these points even when the disclosure is negative.

Sample size	441 participants
Data exclusions	One participant did not meet our eligibility criteria.
Replication	Replication was not necessary in this study design.
Randomization	Not applicable.
Blinding	Not applicable.

Reporting for specific materials, systems and methods

We require information from authors about some types of materials, experimental systems and methods used in many studies. Here, indicate whether each material, system or method listed is relevant to your study. If you are not sure if a list item applies to your research, read the appropriate section before selecting a response.

Materials & experimental systems

n/a	Involved in the study	
☒	☐	Antibodies
☒	☐	Eukaryotic cell lines
☒	☐	Palaeontology and archaeology
☒	☐	Animals and other organisms
☐	☒	Clinical data
☒	☐	Dual use research of concern

Methods

n/a	Involved in the study	
☒	☐	ChIP-seq
☒	☐	Flow cytometry
☒	☐	MRI-based neuroimaging

Clinical data

Policy information about clinical studies

All manuscripts should comply with the ICMJE guidelines for publication of clinical research and a completed CONSORT checklist must be included with all submissions.

Clinical trial registration	Registered at ClinicalTrials.gov Identifier: NCT03096509

Study protocol	The study protocol can be found at: https://pubmed.ncbi.nlm.nih.gov/30882359/
Data collection	Fifty MISSION-ABC community respiratory clinics were held between September 2016 and July 2017.
Outcomes	Clinical outcomes for unscheduled care use, including exacerbations and hospital admissions, and questionnaire responses were compared between the telehealth and control arm.
